# Psychiatric Disorder Criteria and their Application to Research in Different Racial Groups

**DOI:** 10.1186/1471-244X-7-1

**Published:** 2007-01-10

**Authors:** Nancy CP Low, John Hardy

**Affiliations:** 1 Section on Developmental Genetic Epidemiology, National Institute of Mental Health, 35 Convent Drive, MSC 3720, Bethesda, Maryland 20892-3720, USA; 2 Laboratory of Neurogenetics, NIA Intramural Research Program, Porter Neuroscience Building, NIH Main Campus, Bethesda, Maryland 20892, USA

## Abstract

**Background:**

The advent of standardized classification and assessment of psychiatric disorders, and considerable joint efforts among many countries has led to the reporting of international rates of psychiatric disorders, and inevitably, their comparison between different racial groups.

**Results:**

In neurologic diseases with defined genetic etiologies, the same genetic cause has different phenotypes in different racial groups.

**Conclusion:**

We suggest that genetic differences between races mean that diagnostic criteria refined in one racial group, may not be directly and simply applicable to other racial groups and thus more effort needs to be expended on defining diseases in other groups. Cross-racial confounds (in addition to cultural confounds) make the interpretation of rates in different groups even more hazardous than seems to have been appreciated.

## Text

The DSM [1] and ICD [2] psychiatric disorder criteria represent the extracted wisdom of predominantly Caucasian psychiatrists treating predominantly Caucasian patients. They have been useful clinically for the important pragmatic reasons that, within this group, they facilitate efficient communication between physicians about patients, reporting on morbidity and mortality statistics, attempts at common treatments, and billing of third party payers. However, they have little biological validity and there is no evidence that they reflect etiology. They have opened the door to many pharmacologic and behavioral intervention trials [3-6] and etiologic investigations that have, in general, been disappointing. However, these disordered symptom clusters (often comprised of variations in normal human feelings) reflect complexity that we have not been able to fully appreciate and this is in part due to our obscured vision of them through the lens of DSM/ICD classification.

Despite this derivation, cross-racial comparisons of disorder rates are a frequent topic of investigation and interpretation [7,8]. While most researchers would accept that it is challenging to compare diagnostic rates of psychiatric disorders across cultures [9] they have not considered the possibility that clinical manifestations of the same etiology may be different in people of different genetic backgrounds (that is, their race).

While clinical neurological diagnoses are essentially made by the same operational process of clinical consensus, these clinical diagnoses have traditionally been validated through neuropathological examination. In many cases, this biological validation can be made since the genetic etiologies of many neurological diseases are now known. In the limited number of times this biological validation has been compared retrospectively against the clinical (and pathologic) characterization of neurological disease, it has become clear that the clinical characterization holds up poorly in different racial groups. For example, the phenotypes of both Spinocerebellar Ataxia types 2 and 3 (SCA2 and SCA3) are different in Eastern Asians and Sub-Saharan-Africans  respectively from their phenotypes in Caucasians [10-13]. Dentatorubral-Pallidoluysian Atrophy in the Japanese is different from Haw River syndrome in African-Americans despite being caused by the same polyglutamine expansion [14,15]. Presenilin mutations present with Alzheimer's disease with a predominantly early memory change in Caucasians, but seem to present with personality change in those of African descent [16]. These differences in clinical phenotypes are reminiscent of the differences seen in different strains of transgenic mice with the same transgene [17] and should not be surprising to geneticists [18,19]. The process of validation through biological examination is not possible in psychiatric diagnoses (though many attempts are underway in the search for "endophenotypes" or biomarkers); in fact, many psychiatric syndromes are mimicked by known medical disorders, such as, hyper/hypothyroidism, pancreatic cancer, hyperparathyroidism, lyme disease (mood changes, mania, depression); hypo/hyperglycemia, pheochromocytoma (panic), thiamine deficiency, normal pressure hydrocephalus (dementia, cognitive changes), lead poisoning (attention deficit hyperactivity disorder), etc., and a psychiatric diagnosis is only made following their exclusion, as the DSM-IV dictates.

Given this precedent, it seems ill advised to interpret any differences in disorder rates between different racial groups as being indicative of different rates of exposures to risk factors. This potential for confounding genetic variability is in addition to the more widely recognized, culturally derived issues inherent in the interpretation of most "risk factors" in psychiatric disorders, such as parenting styles, low socioeconomic status, temperament, or psychological "trauma." Since the diagnostic criteria have been imperfectly tuned to ensure, as far as possible, that everyone with mental illness in a Caucasian population receives a diagnosis (for billing, treatment, etiologic study and needs assessment surveys), it should not be surprising to find that the rates in non-Caucasians would be different. Therefore, even given identical exposures, one should expect lower rates of DSM/ICD categories in non-Caucasians since less people in those groups would fit comfortably a diagnostic category. The limited data available suggest this is generally the case [18]. This does not imply that the burden of mental distress is less in other races, but rather our instruments of assessment and diagnosis are less appropriately applied in such genetically different individuals.

## Abbreviations

DSM – Diagnostic and Statistical Manual of Mental Disorders; ICD – International Classification of Diseases.

## Competing interests

The author(s) declare that they have no competing interests.

## Authors' contributions

JH and NL conceived of the manuscript. JH drafted the initial version and both participated in multiple revisions until the final version.

## Pre-publication history

The pre-publication history for this paper can be accessed here:


